# Mesoporous Non-stacked Graphene-receptor Sensor for Detecting Nerve Agents

**DOI:** 10.1038/srep33299

**Published:** 2016-09-14

**Authors:** Hee Min Hwang, Eunhee Hwang, Doyoung Kim, Hyoyoung Lee

**Affiliations:** 1 Centre for Integrated Nanostructure Physics (CINAP), Institute of Basic Science (IBS), Department of Energy Science, Sungkyunkwan University, Suwon 440-746, Korea; 2 Centre for Integrated Nanostructure Physics (CINAP), Institute of Basic Science (IBS), Department of Chemistry, Sungkyunkwan University, Suwon 440-746, Korea

## Abstract

A novel gas sensor consisting of porous, non-stacked reduced graphene oxide (NSrGO)-heaxfluorohydoroxypropanyl benzene (HFHPB) nanosheets was successfully fabricated, allowing the detection of dimethyl methyl phosphonate (DMMP), similar to sarin toxic gas. The HFHPB group was chemically grafted to the NSrGO *via* a diazotization reaction to produce NSrGO-HFHPB. The NSrGO-HFHPB 3D film has a mesoporous structure with a large pore volume and high surface area that can sensitively detect DMMP and concurrently selectively signal the DMMP through the chemically-attached HFHPB. The DMMP uptake of the mesoporous NSrGO-HFHPB was 240.03 Hz, 12 times greater than that of rGO-HFHPB (20.14 Hz). In addition, the response rate of NSrGO-HFHPB was faster than that of rGO-HFHPB, an approximately 3 times more rapid recovery due to the mesoporous structure of the NSrGO-HFHPB. The NSrGO-HFHPB sensor exhibited long-term stability due to the use of robust carbon and resulting high resistance to humidity.

Detecting hazardous materials in civilian and military systems has had increasing attention. Chemical warfare agents (CWAs) are highly toxic chemicals that have been used as chemical weapons. Among the CWAs, an organophosphate is a well-known nerve agent that is highly toxic, volatile, and can irreversibly alter enzymes in neurons. Dimethyl methyl phosphonate (DMMP), that has an organophosphate, is a famous simulant for nerve agents, such as sarin and soman, that disrupts the balance of the sympathetic and parasympathetic autonomic nervous system inside DNA^1^,^2^. Due to the structural similarity to sarin and soman, most researchers use DMMP, dimethyl cyanophosphonate (DCNP), or dimethyl chlorophosphonate (DCP) as model compounds for detecting toxic gas vapor materials of CWAs. If humans ingest or are exposed to a high concentration of a 200 mg/m^3^ of sarin nerve agent, death can occur within two minutes^3^. Real cases of the use of nerve agents include the Iran-Iraq war (1980–1998) and the attack on the center of Tokyo in 1990 s by the Aum Shinrikyo sect^4^,^5^,^6^. In these cases, many researchers attempted to detect the nerve agents with the least amount (high sensitivity) of agent and the fastest response. However, problems still remain such as low detecting volume, slow response time, and the need for a complex process^7^,^8^.

To overcome these problems, sensory materials have been developed to fabricate a mesoporous structure (2–50 nm) while maintaining a large uniform pore size with a high surface area to produce a high level of sensor sensitivity. Control of the porous structure has been one of the largest challenges in the manufacture of sensory probes to achieve enhanced sensitivity and a fast response^9^. W. Xu *et al*. fabricated a porous structure sensor consisting of an In_2_O_3_ flower form. In fact, the porous structure sensor that had more active sites showed an approximately 50 times higher response than the flat structure sensor^10^. K. S Chou *et al*. also reported that the sensitivity of a porous ceramic sensor to humidity was greater than that of a film-type ceramic sensor due to the large capacity for water adsorption^11^. These results suggest the porous structure improves the sensitivity. However, in the case of only inorganic mesoporous nanomaterials, problems remain, especially for the long production time, slow response time, high selectivity and sensitivity.

To overcome the sensing limitations of inorganic mesoporous nanomaterials, a porous carbon-based sensor has been investigated as it has unique properties such as a rapid response time and low operating temperature for detecting heavy metals, moisture^12^, or CWA. However, to control the porosity of the carbon-based sensor, porous carbon materials have to be coordinated with other materials, such as metals and metal oxides, or have to be connected to a networking form with a layered structure. Only a few papers have reported on the controlled porous carbon architecture for enhancing sensitivities^13^. J.J. Steele at al. reported a nanostructured gradient index optical filter using a carbon-based material for high-speed humidity sensing. A high-speed response was attained from an unobstructed pore inside gradient films^14^. A. Stein at al. reported porous carbon materials that have large pore volumes are suitable for a fast absorption sensor by introducing K^+^ ions into porous carbon active materials^15^. Thus, based on previous reports, porous structured carbon materials can increase the sensing capacity and also the sensing speed. However, there are still limitations in terms of difficulty controlling the pore size and pore volume using a 1 Dimensional (1D) carbon-based network structure. Therefore, it is necessary to have 2-dimensioanl (2D) carbon-based materials for easy control of pore size and pore volume. To increase the porosity and pore volume of 2D graphene flakes, Yoon *et al*. reported that a non-stacked reduced graphene oxide (NSrGO) has a large surface area and also high pore volume^16^. The surface area (1435.4 m^2^ g^−1^) of the NSrGO powder was 53 times greater than that of other carbon materials (Table S1)^17^,^18^,^19^.

In addition, the use of a good receptor designed for a specific platform has been an important factor for the development of sensing materials. For enhancing the sensitivity and simultaneous selectivity in the sensing system, a precisely designed receptor on the porous materials is still needed. A hexafluorohydoroxypropanyl benzene (HFHPB) compound is one of the best candidates for a receptor of sarin^20^,^21^. HFHPB has an acidic proton of the hydroxy group due to a strong electron-withdrawing trifluoromethly group and is highly sensitive and selective to the organophosphate group of DMMP *via* strong hydrogen-bonding (Fig. 1). However, there has been no report on a 3D porous carbon structure built from porous 2D carbon-based nanosheets anchored with a highly sensitive and selective receptor to CWAs.

Herein, we report a 3D nanostructured porous NSrGO-HFHPB sensor using wrinkled 2D non-stacking reduced graphene oxide (NSrGO) and a HFHPB receptor for effectively detecting DMMP. The HFHPB receptor was chemically attached to the NSrGO *via* diazotization reaction of 2-(4-Aminophenyl)-1,1,1,3,3,3-hexafluoro-2-propanol to produce a new NSrGO-HFHPB compound. The HFHPB receptor consisting of two strong electron withdrawing CF3 groups chemically attached to the NSrGO is a strong Lewis acid and it can uptake a phosphonate group that most nerve agents have *via* hydrogen bonding, expecting to provide a highly selective response only to DMMP vapor even at room temperature^22^. The average pore size of the 3D porous nanostructured NSrGO-HFHPB film is expected to be large enough for DMMP to pass through easily, providing a fast response and also high sensitivity due to the large exposed surface area. To prove the capability of detecting DMMP using a quartz crystal microbalance (QCM), HFHPB-rGO derivatives were prepared including HFHPB-rGO and HFHPB-NSrGO from pristine rGO and pristine NSrGO.

## Materials and Methods

### Synthesis of rGO and NSrGO

Graphene oxide (GO) was prepared by the modified Hummer’s method from natural graphite using sulfuric acid, potassium permanganate, and sodium nitrate. The rGO made by heat treatment using a tube furnace at 1,000 °C for 1 hour (1.5 °C min^−1^). NSrGO was prepared from GO (500 mg) in which the solution was dispersed in ethanol due to full exfoliation of 1 L of the GO solution (5 mg/ml^−1^). After dispersion, 200 ml methyl chloride and 500 ml hexane were added to the GO solution until the GO flakes settled to allow for complete removal of water and the formation of GO sheets. To release, vacuum filtration was applied and the GO sheets were washed with hexane several times. The resulting product was heated by a muffle furnace at 200 °C for 15 min as its surface area dramatically increased. NSrGOs were obtained by thermal treatment using a tube furnace at 1,000 °C for 1 hour (1.5 °C min^−1^) (Fig. S1).

### Synthesis of HFHPB daizonium salts

For the synthesis of HFHPB daizonium salts, 0.9 g 2-(4-aminophenyl)-1,1,1,3,3,3-hexafluoro-2-propanol (2.9 mmol, Alfa Aesar) was dispersed in 10 ml distilled tetrahydrofuran (THF) in a 50 ml round flask and cool to −5 °C. 0.6 ml isoamyl nitrite (4.6 mmol, 1.6 equiv.) was added to the HFHPB derivative solution using a drop wise method and stirred for 10 min. After stirring, 0.72 ml boron trifluoride etherate (5.8 mmol, 2 equiv.) was added to the flask and incubated for 12 h at 60 °C. All steps were performed in a N_2_ atmosphere under an ice bath (−5 °C). The yellow solid was filtered and washed by Et_2_O until it produced a white solid product.

### Synthesis of rGO-HFHPB and NSrGO-HFHPB

Different ratios of NSrGO-HFHPB (1:0.5, 1:4) were synthesized to check the effect of the DMMP sensing properties. For the samples chemically grafted between NSrGO with HFHPB salts, 200 mg NSrGO was dispersed in 200 ml dimethyl formamide with 1 wt% sodium dodecylbenzenesulfonate (SDBS) as a surfactant to maintain the equilibrium of the NSrGO dispersion solution by ultrasonication 1 h. 100 mg of HFHPB salts and 400 mg of controlled ratios of HFHPB 1:0.5, 1:4 with NSrGO) were added to the NSrGO solutions and stirred for 1 day. The resulting solutions were filtered with acetone and dimethyl formamide through a 0.2 μm PTFE membrane (Whatman^TM^) until excess HFIP salts and surfactant were removed. After treatment in a vacuum oven for 1 day at 80 °C, a black sample was obtained. A mass flow controller (MFC) and quartz crystal microbalance (QCM) were used to check the sensing experiment with DMMP flowing at 2 to 64 ppm under 500 ppm of fixed N_2_ gas.

### Material characterizations

The characterizations were confirmed using a JEOL JSM-7404F field emission scanning electron microscopy (SEM) operating at 15 kV, and X-ray photoelectron spectroscopy (XPS) measurements were performed on a Thermo VG Microtech ESCA 2000 with a monochromatic Al-Kα X-ray source at 100 W. Raman spectroscopy measurements were recorded using a Renishaw RM 1000-Invia micro-Raman system with an excitation energy of 2.41 eV (514 nm). The Brunauer-Emmett-Teller (BET) theory was used to determine the specific surface area and the Horvath-Kawazoe (HK) method was used for sub-nanopore analysis via a BELSORP-max. Water contact angle (WCA) analysis was used to confirm the hydrophobicity and hydrophilicity angle. FT-IR spectra were collected using a Thermo Nicolet AVATAR 320 instrument and the powder XRD pattern was acquired using a D8-Advance instrument (Germany) with Cu-Ka radiation. A quartz crystal microbalance (EQCM, Shin) with gold-coated quartz crystal from International Crystal (10 MHz) Manufacturing Co, Inc. was also used.

## Results

For samples, rGO, NSrGO, rGO-HFHPB and NSrGO-HFHPB, were prepared to examine the interaction between DMMP and a sensing platform that is directly influenced by the sensitivity and selectivity to DMMP. First, graphene oxide (GO) was prepared following a modified Hummer’s method from natural graphite. GO was thermally treated at 1,000 degrees to produce reduced graphene oxides (rGOs). Alternatively, GO was treated with nonpolar solvents to produce non-stacked GO (NSGO). The resulting NSGO was reduced by thermal heating to produce NSrGO. The HFHPB receptor was introduced to rGO and NSrGO to produce rGO-HFHPB and NSrGO-HFHPB via a diazotization reaction of rGO and NSrGO, respectively (Supplementary Fig. S1).

### Structural characterization of rGO-HFHPB and NSrGO-HFHPB

Chemical analyses of rGO-HFHPB and NSrGO-HFHPB materials were performed by FT-IR, XPS, XRD and Raman, FT-IR spectra of rGO, rGO-HFHPB, and NSrGO-HFHPB (Fig. S2) illustrated stretching vibration peaks at 1579 cm^−1^ (C=C) and 1353 cm^−1^ (C-H) for the rGO sp^2^ and sp^3^ backbone, respectively. However, the GO peak had a large domain of a broad stretching vibration peak from 3000 to 3600 cm^−1^ for the hydroxyl, carbonyl (C-O/C=O, sp3) and carboxyl (C(O)O) groups while rGO-HFHPB and NSrGO-HFHPB showed a new peak of the hydroxyl group from HFHPB at 3590 cm^−1^, an increase in the C-H stretched bending peak at 3038 cm^−1^ and a new peak at 1197 cm^−1^ for the C-F stretching bond^23^. As expected, the intensity of the C-F stretching peak of NSrGO-HFHPB was greater than that of rGO-HFHPB due to the more exposed surface area of the porous NSrGO film. The XPS data supports the existence of a receptor on its NSrGO-HFHPB platform (Fig. S3). The carbon backbone peak (C-C/C=C, sp^2^) was confirmed at 284.6 eV, indicating the existence of the phenyl peak of HFHPB derivatives. The hydroxyl, carbonyl (C-O/C=O, sp3), and carboxyl (C(O)O) peaks of NSrGO-HFHPB were observed at 285.7, 287.1, and 289.6 eV, respectively. The fluorine (C-F) peak appeared at 292.8 eV and the F1s XPS spectra showed that the C-F peak appeared at 688.4 eV^24^,^25^, indicating that the HFHPB moiety was successfully covalently bonded to the rGO sheets.

The XRD data could also confirm the amorphous structure that usually has a broad peak^26^. In general, the XRD spectrum of the rGO film had a very sharp peak at 2∂ = 26°^27^, while NSrGO has a broad peak at 2∂ = 25° due to a randomly-distributed, non-stacked form of NSrGO nanosheets (Fig. S4).

In addition, the Raman spectra also confirmed the formation of rGO-HFHPB and NSrGO-HFHPB (Fig. S5). Typical peaks of pristine GO existed at 1357 and 1595 cm^−1^ for the D and G peaks, respectively^28^. However, the intensity of the D peaks for rGO-HFHPB and NSrGO-HFHPB increased and shifted toward a lower value (1344 cm^−1^) compared to the rGO peak (1357 m^−1^) as the carbon double bonding (sp^2^) of rGO was hybridized to sp^3^ as the chemical reaction with HFHPB group proceeded. The intensity of the D/G bands (*I*_*D*_/*I*_*G*_) of NSrGO-HFHPB (*ca*.1.06) was greater than that of rGO (*ca*. 0.87) due to the HFHPB attached to NSrGO^29^,^30^. The Raman spectra of NSrGO-HFHPB also provided strong evidence that the HFHPB was chemically attached to the NSrGO surface and its large surface area of NSrGO- HFHPB provided more room to host the HFHPB, creating a more porous 3D structure compared to the NSrGO film.

### Properties of NSrGO and NSrGO-HFHPB

**Figure 2:**
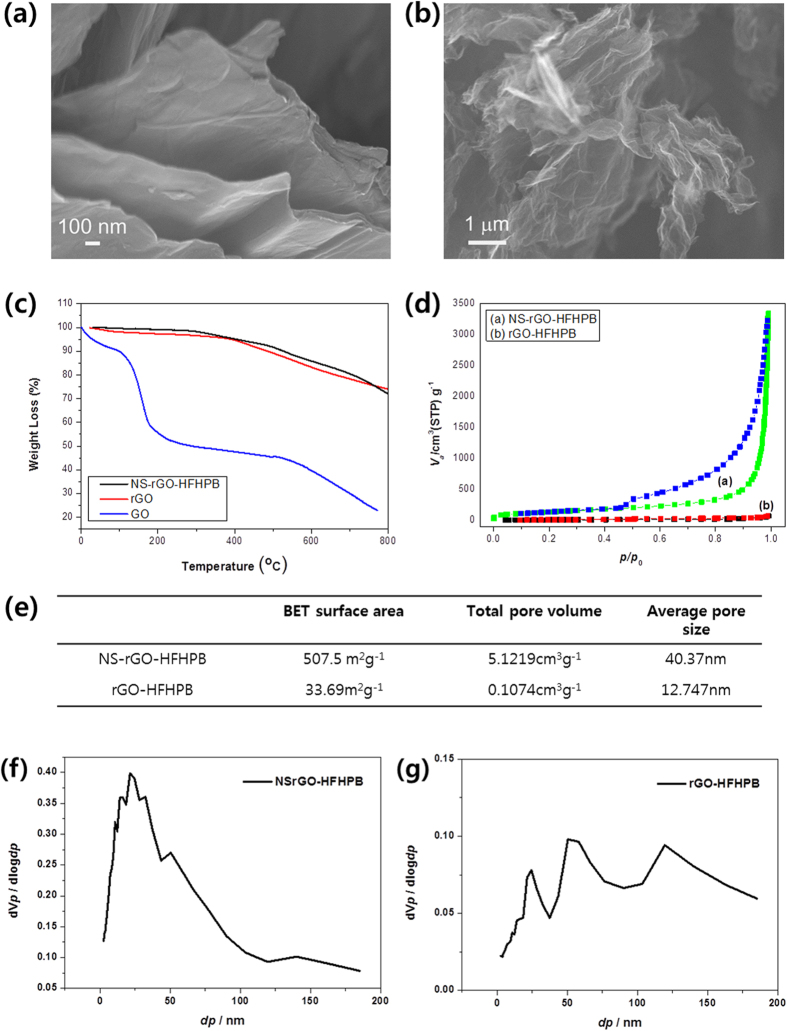
Molecular structure of NSrGO-HFHPB. SEM images of (**a**) rGO-HFHPB and (**b**) NSrGO-HFHPB. TGA curves of (**c**) GO, rGO, and NSrGO-HFHPB. BET data of (**d**) rGO-HFHPB and NSrGO-HFHPB. (**e**) BET table of NSrGO-HFHPB and rGO-HFHPB. Pore size distribution of (**f**,**g**) NSrGO-HFHPB and rGO-HFHPB.

BET and SEM were conducted for investigating the porosity of NSrGO-HFHPB and its sensing properties. Figure 2 shows the SEM images of rGO-HFHPB and NSrGO-HFHPB. The molecular structure of rGO-HFHPB was similar to pristine rGO as shown in Fig. 2a, while that of NSrGO-HFHPB showed a wrinkled structure that can provide the high surface area and high pore volume shown in Fig. 2b. The BET data of absorption and desorption curves are consistent with the SEM results. The TGA curve of GO demonstrates that the weight of GO dramatically reached 50% before 200 °C due to decomposition of the hydroxyl, epoxy, and carbonyl groups as shown in Fig. 2c 31. The TGA curves of NSrGO-HFHPB showed nearly similar behaviors and they slightly decreased from 300 °C with removal of the remaining hydroxyl group in the rGO part. Alternatively, NSrGO-HFHPB retained 71.8% up to 800 °C (Fig. 2c), which is very similar with TGA curve of rGO due to a chemical bonding between NSrGO and HFHPB. This result indicates that the diazotization reaction of NSrGO with HFHPB diazonium salts successfully produced a chemical bond. We confirmed a surface area via BET. The BET method can provide that how many N2 gas can be adsorbed inside of the samples. Blue and green lines are absorption and desorption curves of NSrGO-HFHPB and red and black lines are those of rGO-HFHPB, respectively. The BET curve of NSrGO-HFHPB was changed dramatically due to an increased surface area while that of rGO -HFHPB did not change due to its stacked structure. The surface area of NSrGO-HFHPB (507.5 m^2 ^g^−1^) was 15 times greater than that of rGO-HFHPB (33.69 m^2 ^g^−1^)^32^. The total pore volume (5.122 cm^3 ^g^−1^) was 47.9 times greater than rGO-HFHPB (0.107 cm^3 ^g^−1^) and the average pore size (40.37 nm) of NSrGO-HFHPB was 3.2 times greater than rGO-HFHPB (12.75 nm) (Fig. 2d,e). The Barrertt-Jouner-Halenda method (BJH) was used to measure the pore size distributions of NSrGO-HFHPB and rGO-HFHPB (Fig. 2f,g). The distribution plot of NSrGO-HFHPB provided only one peak at 21.1 nm with a narrow distribution, while that of rGO-HFHPB showed three different peaks such as 25, 60, and 120 nm. The thermal stability of NSrGO-HFHPB compared to GO and rGO was also observed. As stated earlier, rGO was produced by heat treatment at 1,000 °C using a tube furnace.

**Figure 3:**
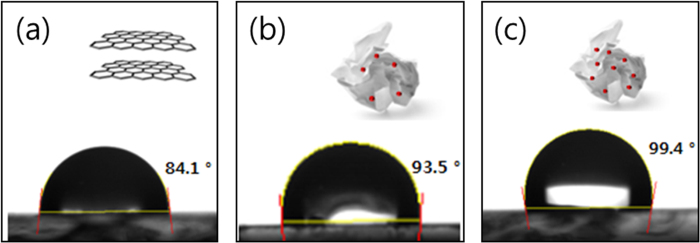
Optical images of the water contact angles of (**a**) GO, (**b**) NSrGO-HFHPB (1:05), and NSrGO-HFHPB (1:4).

The hydrophobicity could also demonstrate that the HFHPB moiety was successfully grafted to NSrGO^33^,^34^. The hydrophobicity of NSrGO-HFHPB was confirmed by water contact angle (WCA) measurements as shown in Fig. 3. Generally, rGO has a much greater hydrophobicity than GO that includes large oxygen functional groups. The fluorines of the CF_3_ group of the HFHPB receptor, which has a low surface free energy even with a hydrophilic hydroxy (−OH) group, sharply increased the hydrophobicity^35^. The higher ratio of the fluorine group *versus* rGO increases the WCA. Specifically, the WCA (99.4°) of NSrGO-HFHPB (1:4) is greater than that (93.5°) of NSrGO-HFHPB (1:0.5) as shown in Fig. 3a–c, confirming that HFHPB was successfully attached to the NSrGO. The advantage of using the high hydrophobic NSrGO-HFHPB is that it can be applied as a humid resistant sensor, even in environmentally harsh conditions.

### Sensing properties of NSrGO and NSrGO-HFHPBs

**Figure 4:**
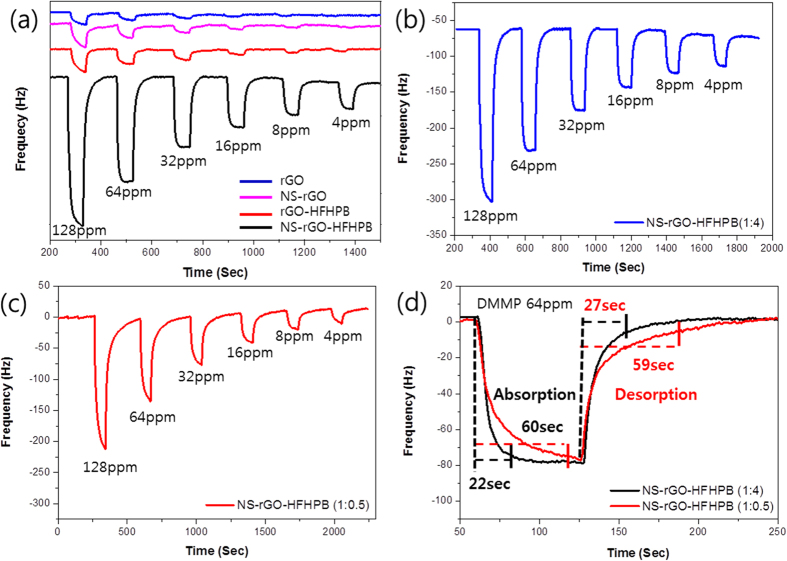
Real response frequency curves of QCM data. Frequencies of (**a**) rGO, NSrGO, rGO-HFHPB and NSrGO-HFHPB under 4 to 128 ppm of DMMP. (**b**,**c**) Frequency curve response of different ratios of NSrGO-HFHPBs. (**d**) Speed of absorption and desorption of different ratios of NSrGO-HFHPB.

The sensing properties of rGO, rGO-HFHPB, NSrGO, and NSrGO-HFHPB were investigated using a quartz crystal microbalance (QCM). The QCM technique has been used widely for mass sensitivity analysis in the sensor field. The QCM method modified with a mass flow controller (MFC) was used to exactly control the amount of DMMP vapor dissolved in various organic solvents^36^,^37^. With absorption and desorption of DMMP vapor on the NSrGO-HFHPB film, the mass changes of the analyte and DMMP were monitored as a frequency on the QCM. Four samples, rGO, rGO-HFHPB, NSrGO, and NSrGO-HFHPB, were prepared on the quartz cell (10 MHz) using a drop casting method on the QCM cell electrode to check the DMMP vapor. The concentrations of the DMMP vapors were 4 to 128 ppm. Nitrogen gas was used as the carrier gas and the flow rate was a fixed at 500 standard cubic centimeters per minute (sccm)^38^. In addition, the average pore size of the 3D porous nanostructured NSrGO-HFHPB film was 40.37 nm, which could easily pass DMMP through a 0.58–0.60 nm pore size^39^,^40^, providing a fast and highly sensitive response due to the large exposed surface area. As shown in Fig. 4a, the rGO sensor detected 128 ppm of DMMP vapor with a 19 Hz frequency, but it did not demonstrate a frequency at a lower concentration of 32 ppm even though a few OH groups on rGO could uptake DMMP via hydrogen bonding. Alternatively, the rGO-HFHPB sensor exhibited a higher frequency than that of the rGO sensor due to a HFHPB receptor, but its sensitivity was not high enough to detect 4 ppm of DMMP vapor. In addition, the porous NSrGO and NSrGO-HFHPB sensors displayed a higher sensitivity than those of the non-porous rGO and rGO-HFHPB sensors. Amazingly, the NSrGO-HFHPB sensor showed the highest sensitivity among the rGO, NSrGO, and rGO-HFHPB sensors with a synergy effect of the porous structure and HFHPB receptor. The NSrGO-HFHPB sensor showed 241, 140, 112, 79, 59, and 43 Hz frequencies at 128 (15.18 × 10^−7 ^g), 64 (8.82 × 10^−8 ^g), 32 (7.05 × 10^−8 ^g), 16 (4.97 × 10^−8 ^g), 8 (3.71 × 10^−8 ^g), and 4 ppm of DMMP vapor (2.70 × 10^−8 ^g), respectively. These frequency values and DMMP ppm on the NSrGO-HFHPB coated quartz crystal cell were calculated as shown in Calculus S1^41^.

**Figure 5:**
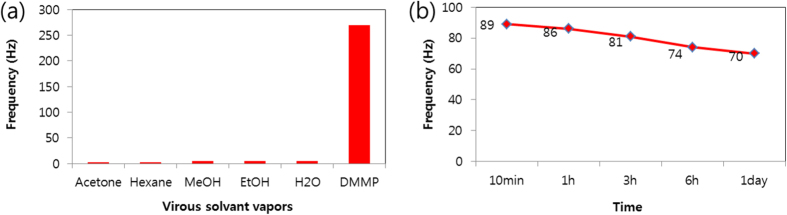
Selectivities and resistance of humidity curves of NSrGO-HFHPB. (**a**) Selectivities of NSrGO-HFHPB with acetone, hexane, MeOH, EtOH, and H_2_O. (**b**) Resistance of humidity of NSrGO-HFHPB over 10 min to 1 day in a humid chamber (humidity: 85%, temperature: 25.5 °C DMMP flow: 64 sccm, N_2_ flow: 500 sccm, time: 60 sec).

In addition, Fig. 4b,c showed two different frequency responses of NSrGO-HFHPB (1:4) and NSrGO-HFHPB (1:0.5) prepared with a NSrGO:HFHPB (1:4) and NSrGO:HFHPB (1:0.5) ratios at 64 ppm of DMMP vapor. The maximum frequency responses showed 83 (1:4) for NSrGO-HFHPB (1:4) and 76 Hz (1:0.5) for NSrGO-HFHPB (1:0.5), respectively. As the ratio of HFHPB *versus* NSrGO increased, the absorption slope increased sharply. Furthermore, the response stabilizing time of the high ratio of HFHPB *versus* NSrGO (approximately 22 s) was 2.7 times shorter than that of the low ratio of HFHPB *versus* NSrGO (approximately 60 s) for 98% of the maximum frequency response to DMMP vapor. The recovery stabilizing time (approximately 27 s) was also 2.2 times shorter, indicating that the HFHPB functional group more effectively catches the DMMP vapor than the enhanced surface area of NSrGO at the 3D porous structure compared to the 3D non-porous structure (Fig. 4a,d). Thus, the NSrGO-HFHPB (1:4) sensor showed the fastest response and recovery to uptake the DMMP vapor. This substrate can be expanded to other poisonous gases. To understand the sensing properties of the NSrGO-HFHPB sensor, various analyzing vapors, such as acetone, methanol, ethanol, hexane, dichloromethane, water and DMMP, were tested under a fixed 500 sccm N_2_ carrier gas (Fig. 5a). EtOH, MeOH, and water vapors that have OH groups showed higher sensitivity than acetone and hexane, but their sensitivity compared to DMMP vapor was nearly negligible, which meant that NSrGO-HFHPB was the perfect material to selectively sense the DMMP vapor.

Finally, the humidity resistance of the NSrGO-HFHPB sensor was investigated from 10 min to 1 day with a homemade humidity system (Fig. S5) at 85% humidity and 25.5 °C temperature. After a moisture treatment, 500 sccm of nitrogen gas was used as a carrier gas with 64 ppm of DMMP vapor flowing. The frequency responses of the NSrGO-HFHPB gradually decreased from 89 Hz after 10 min to 70 Hz after 1 day, indicating that the frequency response even after 1 day in the humid chamber did not change significantly due to its hydrophobic property (Figs 5b, S6 and S7), suggesting that the chemically-modified mesoporous NSrGO-HFHPB film can effectively detect nerve agents and guide a new direction for carbon-based sensor devices.

## Discussion

In summary, we successfully prepared a new mesoporous NSrGO-HFHPB compound *via* a diazotization reaction of NSrGO sheets with a HFHPB receptor and its 3D carbon nanostructured film. The resulting, mesoporous NSrGO-HFHPB film showed a high surface area (507.5 m^2 ^g^−1^), high pore size (40.37 nm), and high pore volume (5.1219 cm^3 ^g^−1^). The mesoporous NSrGO-HFHPB film structure with a high ratio of HFHPB receptors produced a largely exposed contact area and provided a fast response for receptor-target interactions inside the interlayers without obstruction. The NSrGO-HFHPB film sensor selectively detected only phosphonate nerve agents among other solvents including alcohol groups. As a result, the novel NSrGO-HFHPB sensor had 13 times greater sensitivity to DMMP vapors than rGO. As the amount of the HFHPB receptor increased, NSrGO-HFHPB was 3 times faster and had a 2 times shorter recovery, which shows a synergetic effect of the mesoporous structure and receptor. With the help of hydrophobic HFHPB receptors, the NSrGO-HFHPB hydrophobic sensor sustained even environmentally harsh humid conditions, maintaining the sensing capability at 70% in humidity. The new NSrGO-HFHPB sensor system will guide promising strategies of chemical engineering methods for embedding molecules on diverse carbon-based platforms.

## Additional Information

**How to cite this article**: Hwang, H. M. *et al*. Mesoporous Non-stacked Graphene-receptor Sensor for Detecting Nerve Agents. *Sci. Rep.* **6**, 33299; doi: 10.1038/srep33299 (2016).

## Supplementary Material

Supplementary Information

## Figures and Tables

**Figure 1:**
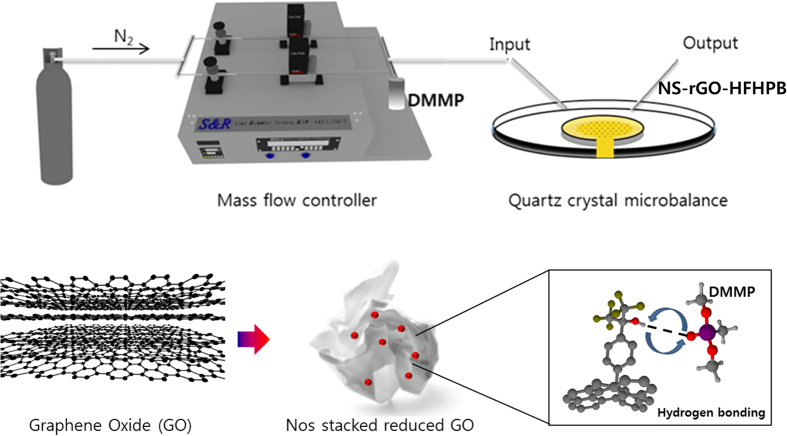
Illustration of the molecular structure of non-stacked, grafted reduce graphene oxide with HFHPB derivative uptake of DMMP.
