# Supplementary material for: Mesoporous Non-stacked Graphene-receptor Sensor for Detecting Nerve Agents
Source: Sci Rep. 2016 Sep 14;6:33299. doi: 10.1038/srep33299 (PMC5022036; doi:10.1038/srep33299)
Supplement: Supplementary Information [file srep33299-s1.doc]

Supplementary Information

**Mesoporous Non-stacked Graphene-receptor**

**Sensor for Detecting Nerve Agents**

*Hee Min Hwang, Eunhee Hwang, Doyoung Kim & Hyoyoung Lee**

H. Hwang, D. Kim, Prof. H. Lee

Centre for Integrated Nanostructure Physics (CINAP), Institute of Basic Science (IBS), Department of Energy Science, Sungkyunkwan University, Suwon 440-746. Korea.

E-mail : hyoyoung@skku.edu; Fax: (+) 82-31-299-5934; Tel: (+) 82-31-299-4566

E. Hwang, Prof. H. Lee

Centre for Integrated Nanostructure Physics (CINAP), Institute of Basic Science (IBS), Department of Chemistry, Sungkyunkwan University, Suwon 440-746. Korea


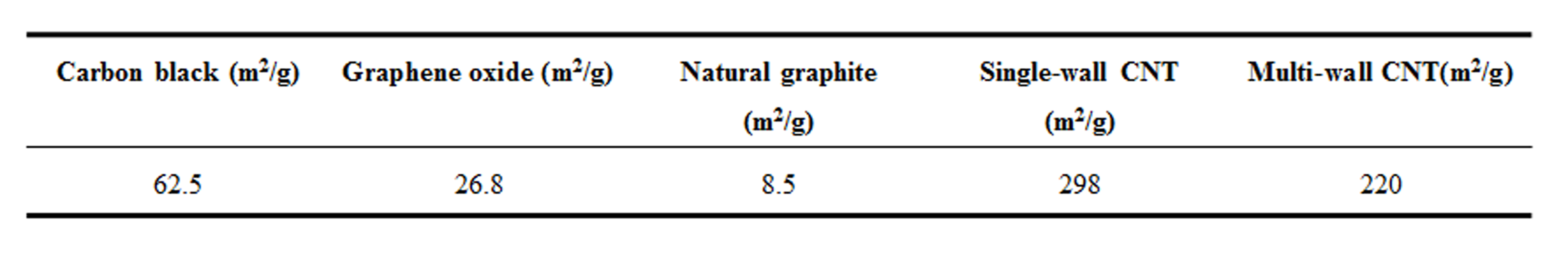


**Table S1.** Specific surface area of the carbon materials


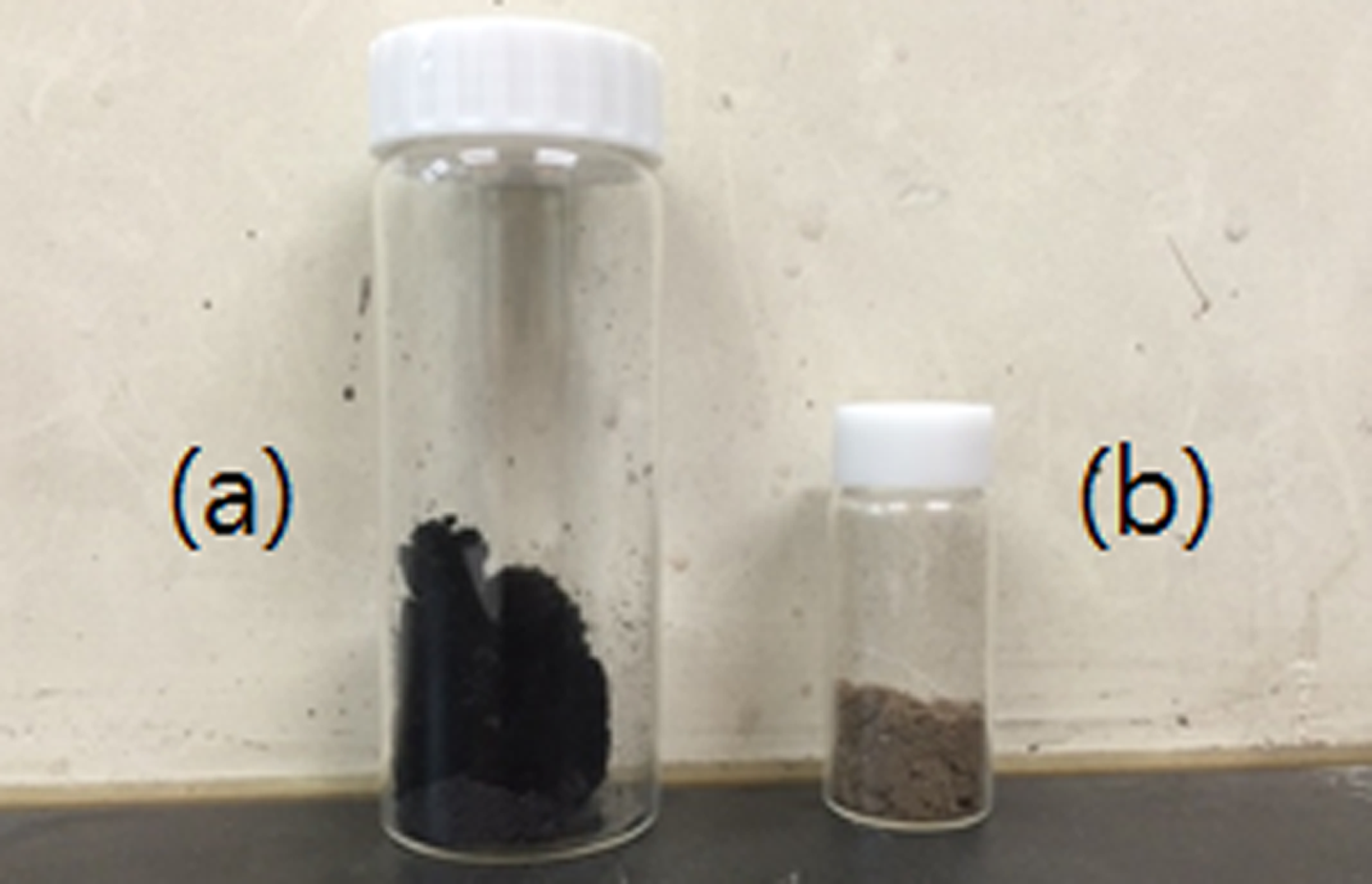


**Figure S1.** (a) Non-stacked reduced graphene and (b) pristine graphene oxide. The amount used was 50 mg.


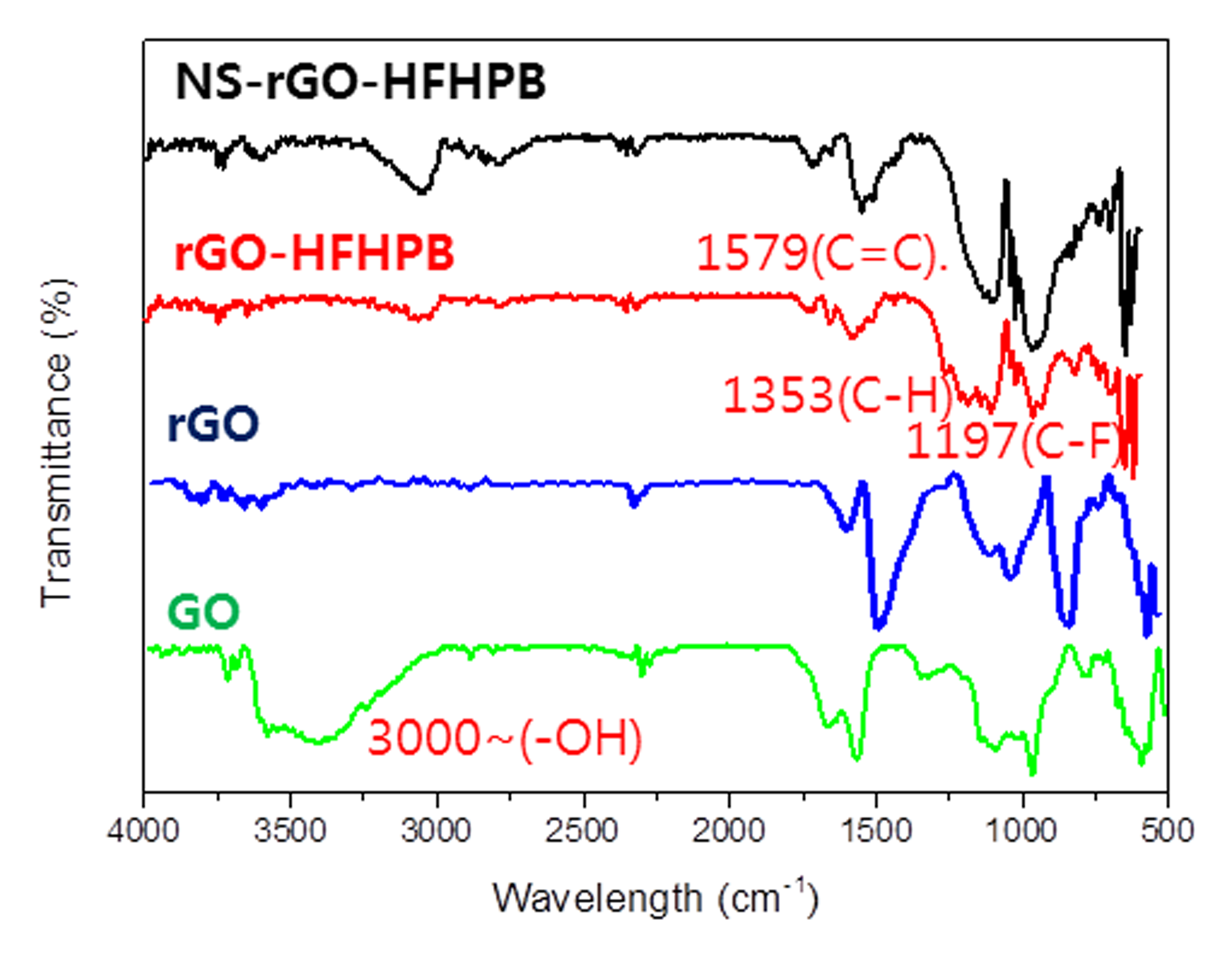


**Figure S2.** FT-IR spectra of GO, rGO, rGO-HFIP, and NSrGO-HFIP.


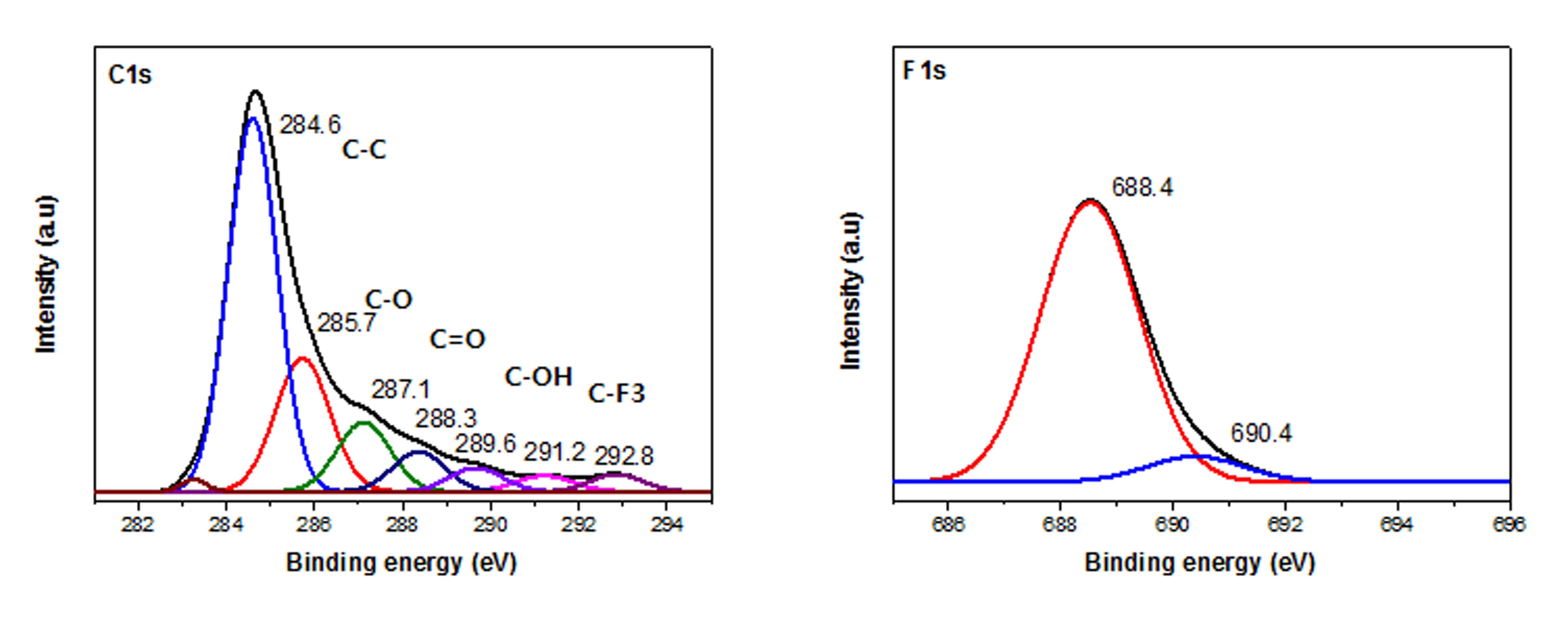


**Figure S3.** XPS data of NSrGO-HFHPB.

**
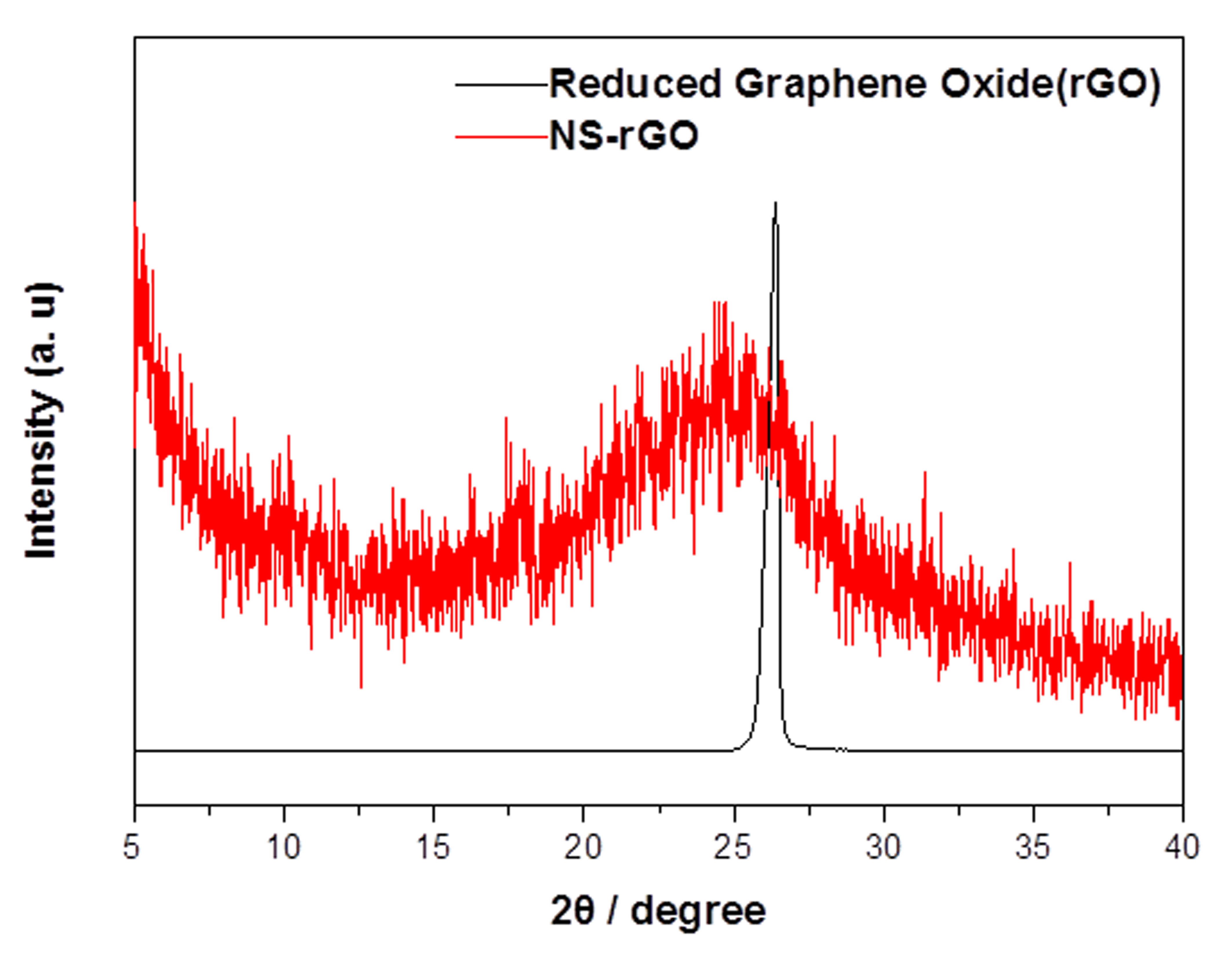
**

**Figure S4.** X-ray diffraction (XRD) spectra of rGO and NSrGO.

**
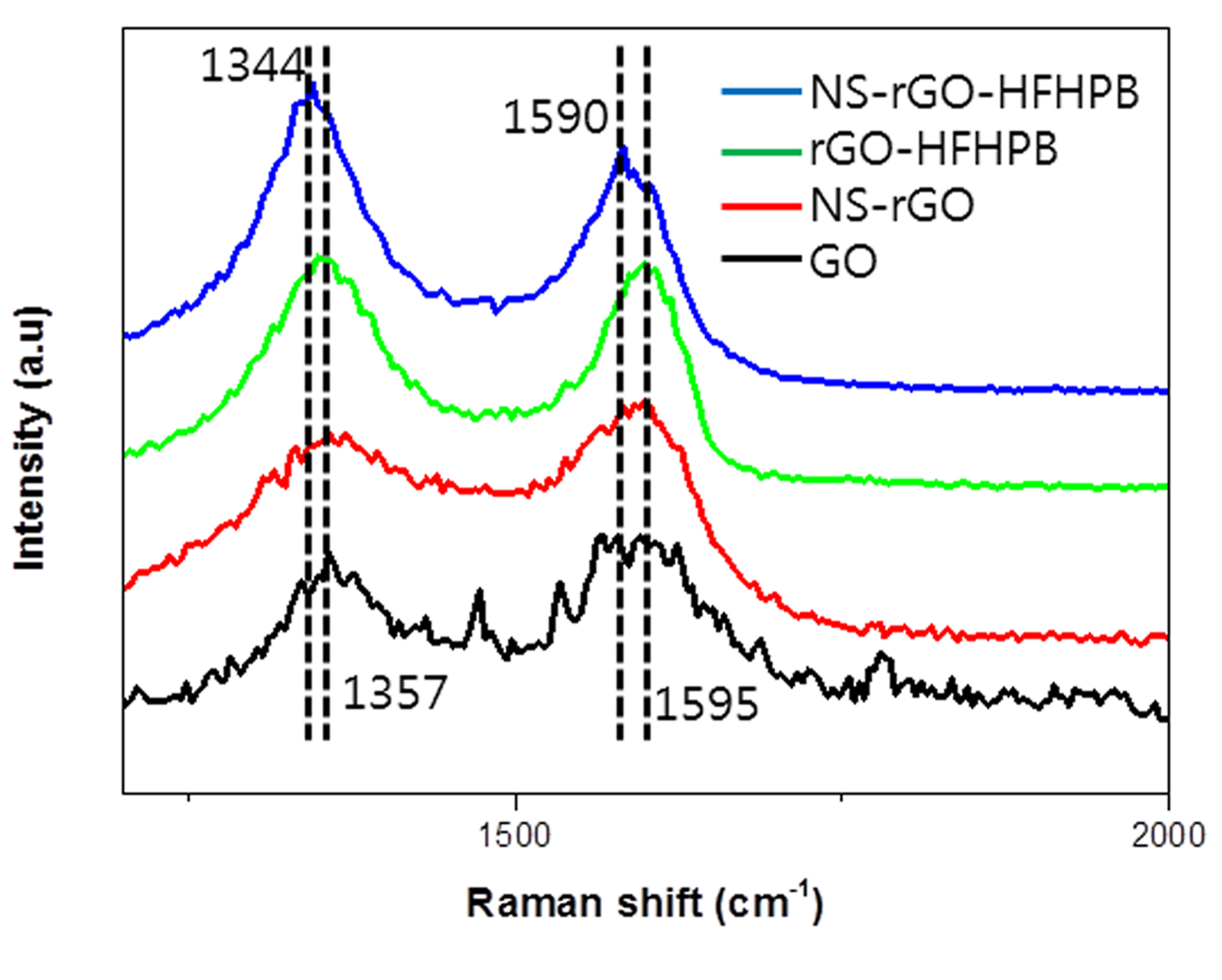
**

**Figure S5.** Raman spectra of GO, rGO-HFHPB, NSrGO and NSrGO-HFHPB.

Nitrogen gas was used as the carrier gas of DMMP through a bubbling system. The sccm unit was converted to ppm and used in the following formula (1).


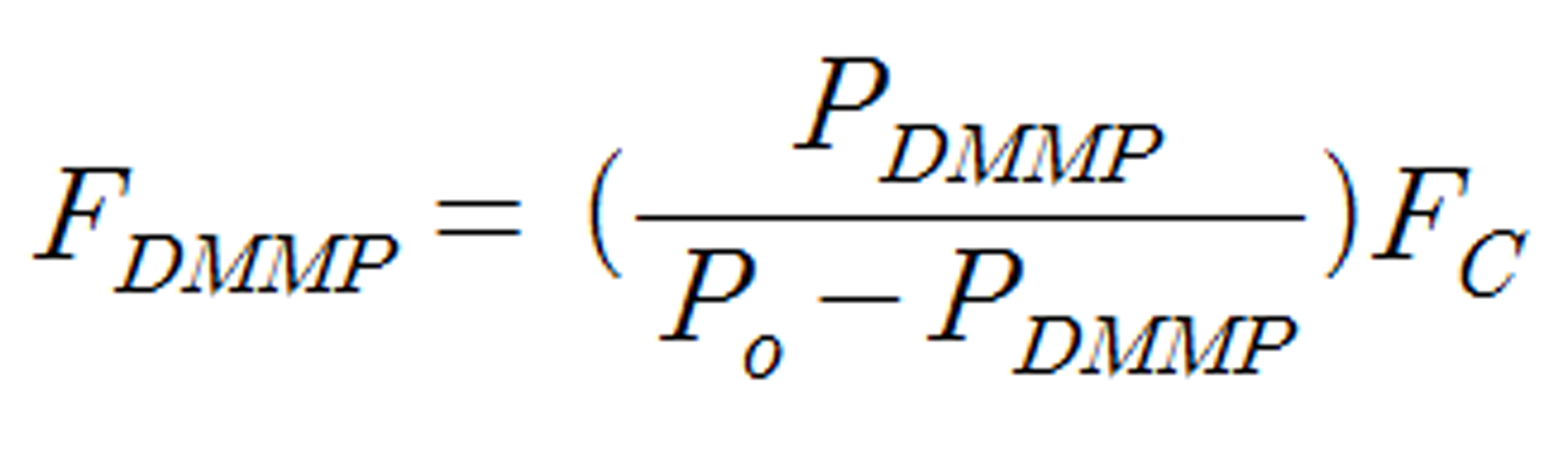
 (1)

The flow rate of DMMP (*F*_DMMP_) was (output pressure of DMMP (P_DMMP_)/outlet pressure of DMMP chamber (P_0_)-output pressure of DMMP (P_DMMP_)(mmHg)) and F_C_ was the rate of carrier gas (N_2_).


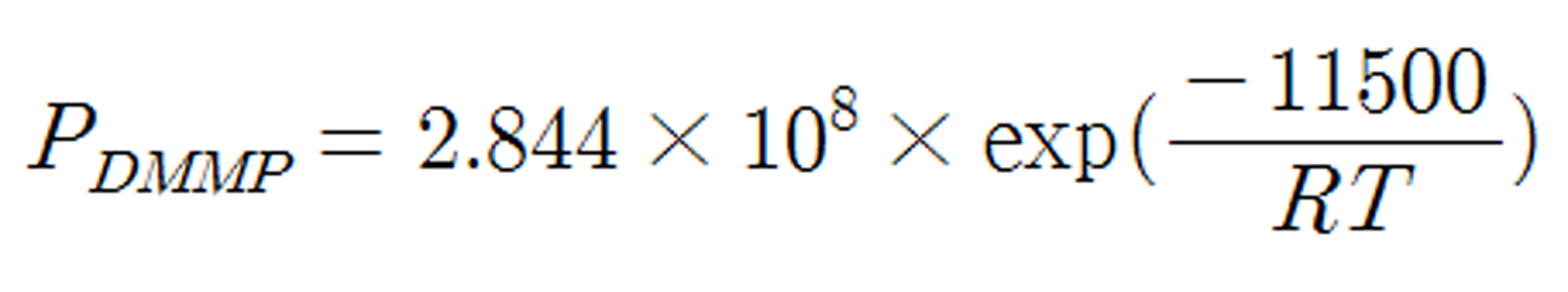
 (2)

*T* (K) is the temperature of DMMP in a bubbler chamber and the vapor pressure was 112 Pa.

The value of the frequency change were calculated from the Sauerbrey equation,


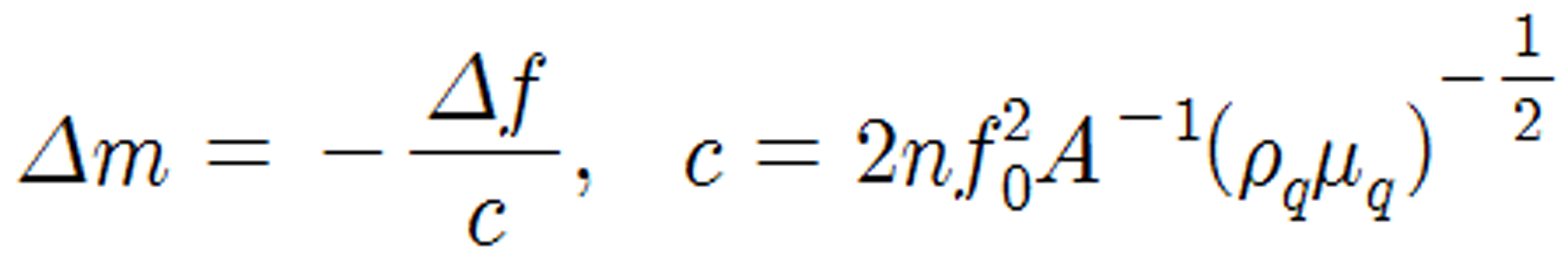


In this formula, the harmonic (n) was represented by 1, resonant frequency (ƒ_0_) was the hertz of the quartz cell (10 MHz), the size of active area on cell (A) was 0.28 cm^2^, the density (
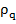
) was 2.648 g cm^-3^ and the shear modulus of quartz for the AT-cut crystal (μq) was (2.947 x 1011 g cm^-1^ s^-2^) producing a Δm = - 0.63 x 10^-9^ x Δƒ.

**Calculus S1.** Unit conversion and frequency calculation.


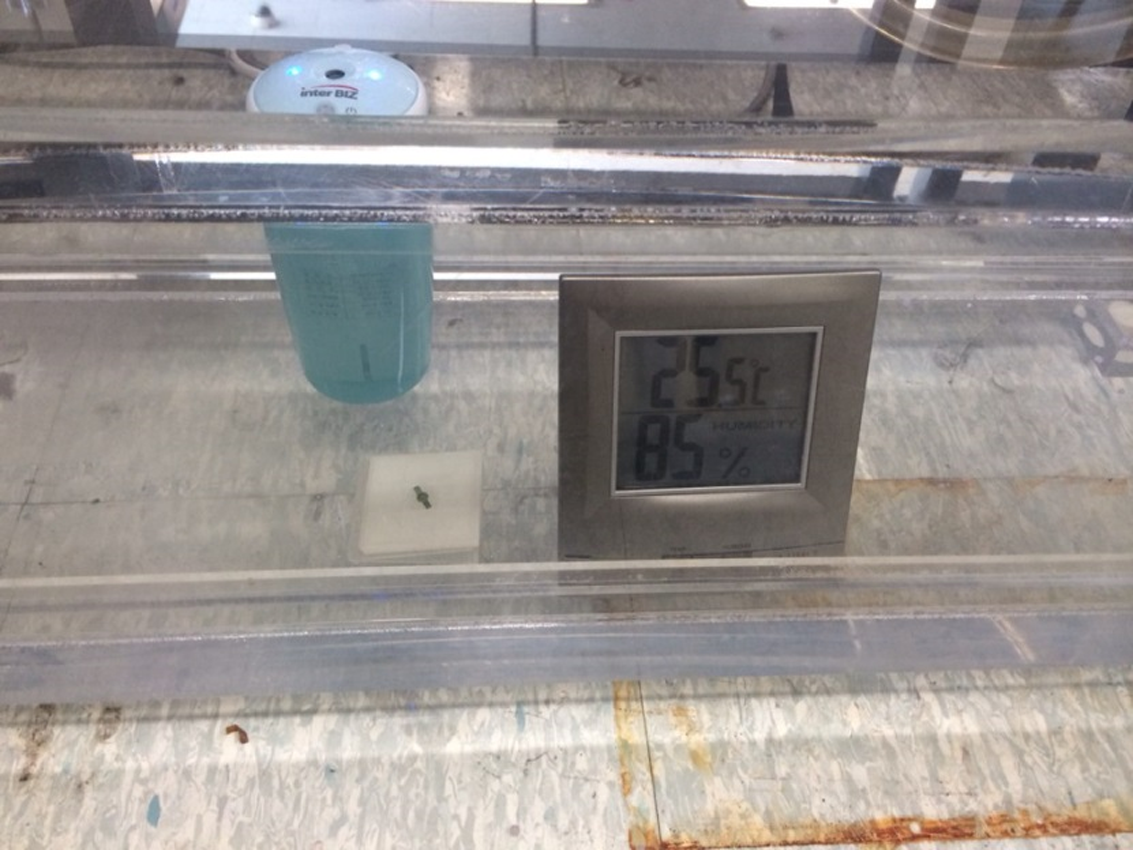


**Figure S6.** Homemade humidity test system (humidity : 85 %, temperature : 25.5 ℃).

**
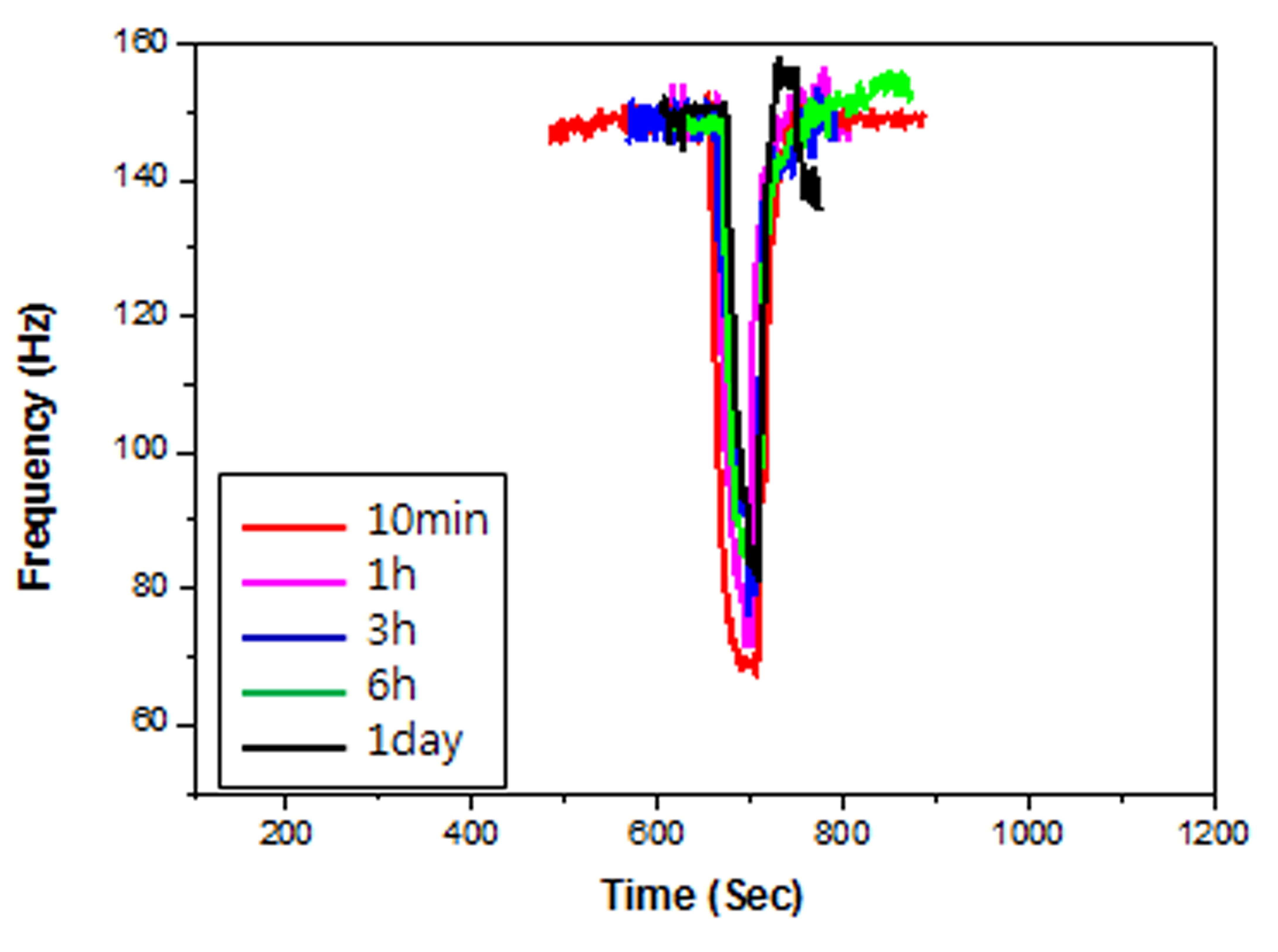
**

**Figure S7.** Real response of the humid resistance frequency of NSrGO-HFIP from 10 min to 1 day.
